# ENLIVE: An Efficient Nonlinear Method for Calibrationless and Robust Parallel Imaging

**DOI:** 10.1038/s41598-019-39888-7

**Published:** 2019-02-28

**Authors:** H. Christian M. Holme, Sebastian Rosenzweig, Frank Ong, Robin N. Wilke, Michael Lustig, Martin Uecker

**Affiliations:** 1 Institute for Diagnostic and Interventional Radiology, University Medical Center Göttingen, Göttingen, Germany; 2 German Centre for Cardiovascular Research (DZHK), Partner site Göttingen, Göttingen, Germany; 3 Departement of Electrical Engineering and Computer Sciences, University of California, Berkeley, USA

## Abstract

Robustness against data inconsistencies, imaging artifacts and acquisition speed are crucial factors limiting the possible range of applications for magnetic resonance imaging (MRI). Therefore, we report a novel calibrationless parallel imaging technique which simultaneously estimates coil profiles and image content in a relaxed forward model. Our method is robust against a wide class of data inconsistencies, minimizes imaging artifacts and is comparably fast, combining important advantages of many conceptually different state-of-the-art parallel imaging approaches. Depending on the experimental setting, data can be undersampled well below the Nyquist limit. Here, even high acceleration factors yield excellent imaging results while being robust to noise and the occurrence of phase singularities in the image domain, as we show on different data. Moreover, our method successfully reconstructs acquisitions with insufficient field-of-view. We further compare our approach to ESPIRiT and SAKE using spin-echo and gradient echo MRI data from the human head and knee. In addition, we show its applicability to non-Cartesian imaging on radial FLASH cardiac MRI data. Using theoretical considerations, we show that ENLIVE can be related to a low-rank formulation of blind multi-channel deconvolution, explaining why it inherently promotes low-rank solutions.

## Introduction

Since acquisition speed is a major issue in MRI, accelerated imaging with multiple receiver coils has been an active field of research since its beginning. Quite rapidly, two main categories of parallel imaging methods emerged: image-space methods, of which sensitivity encoding (SENSE)^1^ is the prototypical example and k-space methods, where it is generalized autocalibrating partially parallel acquisitions (GRAPPA)^2^. SENSE-like methods, when the coil sensitivity profiles are known, permit a natural description as a linear inverse problem. Incorporating the estimation of coil sensitivity profiles into the reconstruction leads to a nonlinear inverse problem, as formulated in Joint Image Reconstruction and Sensitivity Estimation in SENSE (JSENSE)^3^ and Regularized Nonlinear Inversion (NLINV)^4^.

Additionally, low-rank and subspace methods^5–8^ have been proposed to further increase reliability and acceleration in MRI. These methods exploit prior knowledge on the structure of the matrices arising in MRI reconstruction. Recently, ESPIRiT^9^ has been shown to provide robustness towards data inconsistencies similar to k-space methods such as GRAPPA^2^. In particular, in cases where the chosen field-of-view (FOV) is smaller than the object^10^ and in phase-constraint imaging^11^, it was shown that methods based on traditional SENSE that only use a single set of coil sensitivity profiles exhibit artifacts. In ESPIRiT, robust reconstruction is possible through a relaxed SENSE-model, which uses multiple images and sets of coil sensitivity profiles.

ESPIRiT recovers accurate coil sensitivities using an eigenvalue decomposition of an image-domain operator which projects onto the signal space of the calibration matrix. In case of inconsistencies, it produces multiple sets of maps which can be used in a relaxed SENSE reconstruction. ESPIRiT requires a fully-sampled calibration region in the center of k-space. Additionally, it cannot be applied directly to non-Cartesian data, requiring an additional gridding step to generate calibration data. A more generic subspace method is SAKE^5^, because it can be directly applied to data without fully-sampled calibration region or non-Cartesian data. Based on the idea that the signal is contained in a sub-space of smaller dimensionality which can be recovered, SAKE uses structured low-rank matrix completion to recover a full k-space from incomplete data. Unfortunately, it is computationally extremely demanding as each iteration has to perform a singular-value decomposition (SVD). Furthermore, because it operates completely in k-space, regularization terms may require additional Fourier transforms and must be applied to all channels. Calibration-free locally low-rank encouraging reconstruction (CLEAR)^8^ is a related method which uses local low-rankness in the image domain instead of the global k-space rank penalty used in SAKE. This reduces the computational complexity by reducing the size of the needed SVDs, although it does increase the number of SVDs necessary. Furthermore, as it is an image space method, regularization can be integrated more easily.

Regularized Nonlinear Inversion (NLINV)^4^ jointly estimates the image content and the coil sensitivity profiles using a nonlinear algorithm. Similar to SAKE, it does not require a fully-sampled Cartesian calibration region and can be applied directly to non-Cartesian data.

This work aims at combining the advantages from these different methods. Inspired by ESPIRiT, we propose an extension to NLINV that extends it beyond the original SENSE-like model. This method, termed ENLIVE (Extended NonLinear InVersion inspired by ESPIRiT), can be related to a convex relaxation of the NLINV problem subject to a low-rank constraint. From NLINV, it inherits its flexibility and suitability for calibrationless and non-Cartesian imaging; from ESPIRiT it inherits robustness to data inconsistencies. We apply ENLIVE to several imaging settings covering limited FOV, phase constraints, phase singularities, and non-Cartesian acquisition. Additionally, we present comparisons to ESPIRiT and to SAKE.

Initial results have been presented at the 25th Annual Meeting of the International Society for Magnetic Resonance in Medicine^12^.

## Theory

### Formulation

NLINV recovers the image ***m*** and the coil sensitivity profiles ***c***_*j*_ from measurements ***y***_*j*_ by solving the regularized nonlinear optimization problem:

1 $$\mathop{{\rm{\arg }}\,{\rm{\min }}}\limits_{{\boldsymbol{m}},{{\boldsymbol{c}}}_{j}}\,{\sum }_{j=1}^{{N}_{C}}{\Vert {{\boldsymbol{y}}}_{j}-{\mathscr{P}} {\mathcal F} \{{{\boldsymbol{c}}}_{j}\odot {\boldsymbol{m}}\}\Vert }_{2}^{2}+\alpha ({\sum }_{j=1}^{{N}_{C}}{\Vert {\boldsymbol{W}}{{\boldsymbol{c}}}_{j}\Vert }_{2}^{2}+{\Vert {\boldsymbol{m}}\Vert }_{2}^{2})$$

with *N*_*C*_ coils, the two or three dimensional Fourier transform ${\mathcal F}$, the projection ${\mathscr{P}}$ onto the measured trajectory (or the acquired pattern in Cartesian imaging) and an invertible weighting matrix ***W*** penalizing high frequencies in the coil profiles. Here, both image ${\boldsymbol{m}}\in {{\mathscr{C}}}^{{n}_{x}\cdot {n}_{y}\cdot {n}_{z}}$ and coils ${{\boldsymbol{c}}}_{j}\in {{\mathscr{C}}}^{{n}_{x}\cdot {n}_{y}\cdot {n}_{z}}$ are regarded as vectors of size ${n}_{x}\cdot {n}_{y}\cdot {n}_{z}=\,:{N}_{I}$ and $\odot$ is their element-wise product.

In this work, we propose to extend this model to:

2 $$\mathop{{\rm{\arg }}\,{\rm{\min }}}\limits_{{{\boldsymbol{m}}}^{i},{{\boldsymbol{c}}}_{j}^{i}}\,{\sum }_{j=1}^{{N}_{C}}{\Vert {{\boldsymbol{y}}}_{j}-{\mathscr{P}} {\mathcal F} \{{\sum }_{i=1}^{k}{{\boldsymbol{c}}}_{j}^{i}\odot {{\boldsymbol{m}}}^{i}\}\Vert }_{2}^{2}+\alpha \,{\sum }_{i=1}^{k}({\sum }_{j=1}^{{N}_{C}}{\Vert {\boldsymbol{W}}{{\boldsymbol{c}}}_{j}^{i}\Vert }_{2}^{2}+{\Vert {{\boldsymbol{m}}}^{i}\Vert }_{2}^{2})$$

where ${{\boldsymbol{c}}}_{j}^{i}$ and ***m***^*i*^ are *k* sets of unknown coil sensitivity profiles and unknown images. This approach is inspired by ESPIRiT, which uses additional maps to account for model violations^9^.

In the following, we will show that this formulation automatically produces solutions with rank even smaller than *k* if one exits. To show this, we first relate Eq. (2) to a linear inverse problem for matrices with nuclear norm regularization.

From here on, we assume that the variable transformation ${\hat{{\boldsymbol{c}}}}_{j}={\boldsymbol{W}}{{\boldsymbol{c}}}_{j}$ has been applied to move the weighting matrix from the regularization into the forward operator. We note that this problem is equivalent to a corresponding multi-channel blind deconvolution problem^13^ in k-space via the convolution theorem. Using the “lifting” approach used for such blind deconvolution problems^14^, which can also be applied in the image domain, we now lift the Eq. (1) into a linear inverse problem in terms of a rank-1 matrix ***X*** = ***uv***^*T*^ formed by the tensor product of ***u*** and ***v***, where ***u*** corresponds to ***m*** and ***v*** is a stacked vector composed of the weighted coil sensitivity profiles ${\hat{{\boldsymbol{c}}}}_{j}$. The problem then becomes:

3 $$\mathop{{\rm{\arg }}\,\,{\rm{\min }}}\limits_{{\boldsymbol{u}},{\boldsymbol{v}}}\,{\Vert y-{\mathscr{A}}\{{\boldsymbol{u}}{{\boldsymbol{v}}}^{T}\}\Vert }_{2}^{2}+\alpha ({\Vert {\boldsymbol{u}}\Vert }_{2}^{2}+{\Vert {\boldsymbol{v}}\Vert }_{2}^{2})$$

with a linear operator ${\mathscr{A}}$ mapping ***uv***^*T*^ to ${\mathscr{P}} {\mathcal F} {{\boldsymbol{c}}}_{j}\odot {\boldsymbol{m}}$ and a vector ***y*** containing measurement data of all coils. Such an ${\mathscr{A}}$ exists because ***uv***^*T*^ contains all possible products of elements of ***u*** and ***v***. Its explicit action is explained in more detail in the Appendix. In general, all bilinear functions can be expressed as linear functions on the tensor product of the two vector spaces involved.

As suggested by Ahmed *et al*.^14^ for blind multi-channel deconvolution, we now relax the rank-1 constraint and allow *k* sets of images and coil sensitivity profiles. This corresponds to using ${\boldsymbol{X}}={\boldsymbol{U}}{{\boldsymbol{V}}}^{T}\in {{\mathscr{C}}}^{{N}_{I}\times {N}_{C}\cdot {N}_{I}}$ with ${\boldsymbol{U}}\in {{\boldsymbol{C}}}^{{N}_{I}\times k}$ and ${\boldsymbol{V}}\in {{\mathscr{C}}}^{{N}_{C}\cdot {N}_{I}\times k}$, which then leads to the optimization problem

4 $$\mathop{{\rm{\arg }}\,\,{\rm{\min }}}\limits_{{\boldsymbol{U}},{\boldsymbol{V}}}\,{\Vert {\boldsymbol{y}}-{\mathscr{A}}\{{\boldsymbol{U}}{{\boldsymbol{V}}}^{T}\}\Vert }_{2}^{2}+\alpha ({\Vert {\boldsymbol{U}}\Vert }_{F}^{2}+{\Vert {\boldsymbol{V}}\Vert }_{F}^{2})$$

with the Frobenius norm ${\Vert \cdot \Vert }_{F}$. In the Appendix we show how this corresponds to ENLIVE as formulated in Eq. (2). Under conditions given below, Eq. (4) is equivalent to a convex optimization problem for the matrix

5 $$\mathop{{\rm{\arg }}\,\,{\rm{\min }}}\limits_{{\boldsymbol{X}}}\,{\Vert {\boldsymbol{y}}-{\mathscr{A}}\{{\boldsymbol{X}}\}\Vert }_{2}^{2}+2\alpha {\Vert {\boldsymbol{X}}\Vert }_{\ast }$$

with nuclear norm ${\Vert \cdot \Vert }_{\ast }$ regularization^15,16^. The nuclear norm promotes low-rank solutions. Furthermore, if the solution to Eq. (5) has rank smaller than or equal to *k* both problems are equivalent in the sense that from a solution ***U***, ***V*** of Eq. (4) one obtains a solution of Eq. (5) via ***X*** = ***UV***^*T*^ which attains the same value and from a solution ***X*** of Eq. (5) one can construct a solution of Eq. (4) that attains the same value. This is achieved by factorizing ***X*** using the SVD and by distributing the singular values in an optimal way, i.e. equally as square roots, to the two factors. Please note that we do not propose to use this convex formulation for computation as it is very expensive, instead we propose to use the nonlinear formulation given in Eq. (2). Nevertheless, this relationship to nuclear-norm regularization is important as it explains why ENLIVE produces solutions with low rank even smaller than *k*, if one exists.

### Implementation

Similar to NLINV^4^, we solve Eq. (2) using the iteratively regularized Gauss-Newton method (IRGNM). The IRGNM solves successive linearizations with the regularization parameter decreasing in each Newton step: Starting from *α*_0_, the regularizations in each step is reduced according to *α*_*n*_ = *α*_0_*q*^*n*−1^, 0 < *q* < 1. As initial guess, we use ***m***^*i*^ ≡ 1 for the images and ${{\boldsymbol{c}}}_{j}^{i}\equiv 0$ for the coil sensitivity profiles. Because we initialize images and sensitivity profiles for all sets in the same way, the problem is symmetric in the sets and the algorithm will produce degenerate solutions with identical sets. To break this symmetry, we require the *k* sets of coil profiles to be orthogonal using Gram-Schmidt orthogonalization after each Newton step. For orthogonalization, the coil profiles of each set are treated as stacked one-dimensional vectors.

The weighting matrix ***W*** enforcing smoothness in the coil profiles was chosen as in^4^. In k-space, this leads to a penalty increasing with distance from the center of k-space according to ${\mathrm{(1}+a{\Vert {\boldsymbol{k}}\Vert }^{2})}^{b\mathrm{/2}}$. In this work, *a* = 240 and *b* = 40 were used. Furthermore, k-space is normalized so that it extends from −*n*_*i*_/2 to *n*_*i*_/2 for *i* ∈ {*x*, *y*, *z*}. As ***W*** applies weights in k-space, it is the product of a Fourier matrix transforming each coil profile to k-space an of this diagonal weight matrix.

Images and coil profiles are combined in a post-processing step. This is used to either create individual images for each set *i* by combining coil-weighted images ${{\boldsymbol{m}}}^{i}{{\boldsymbol{c}}}_{j}^{i}$ using

6 $${{\boldsymbol{M}}}^{i}=\sqrt{\sum _{j=1}^{{N}_{C}}|{{\boldsymbol{m}}}^{i}\odot {{\boldsymbol{c}}}_{j}^{i}{|}^{2}}$$

or to create a single combined image by first combining each set to obtain a proper image for each coil and then doing a final coil combination with

7 $${\boldsymbol{M}}=\sqrt{\sum _{j=1}^{{N}_{C}}|\sum _{i=1}^{k}{{\boldsymbol{m}}}^{i}\odot {{\boldsymbol{c}}}_{j}^{i}{|}^{2}}\mathrm{.}$$

## Results

### Limited FOV

**Figure 1:**
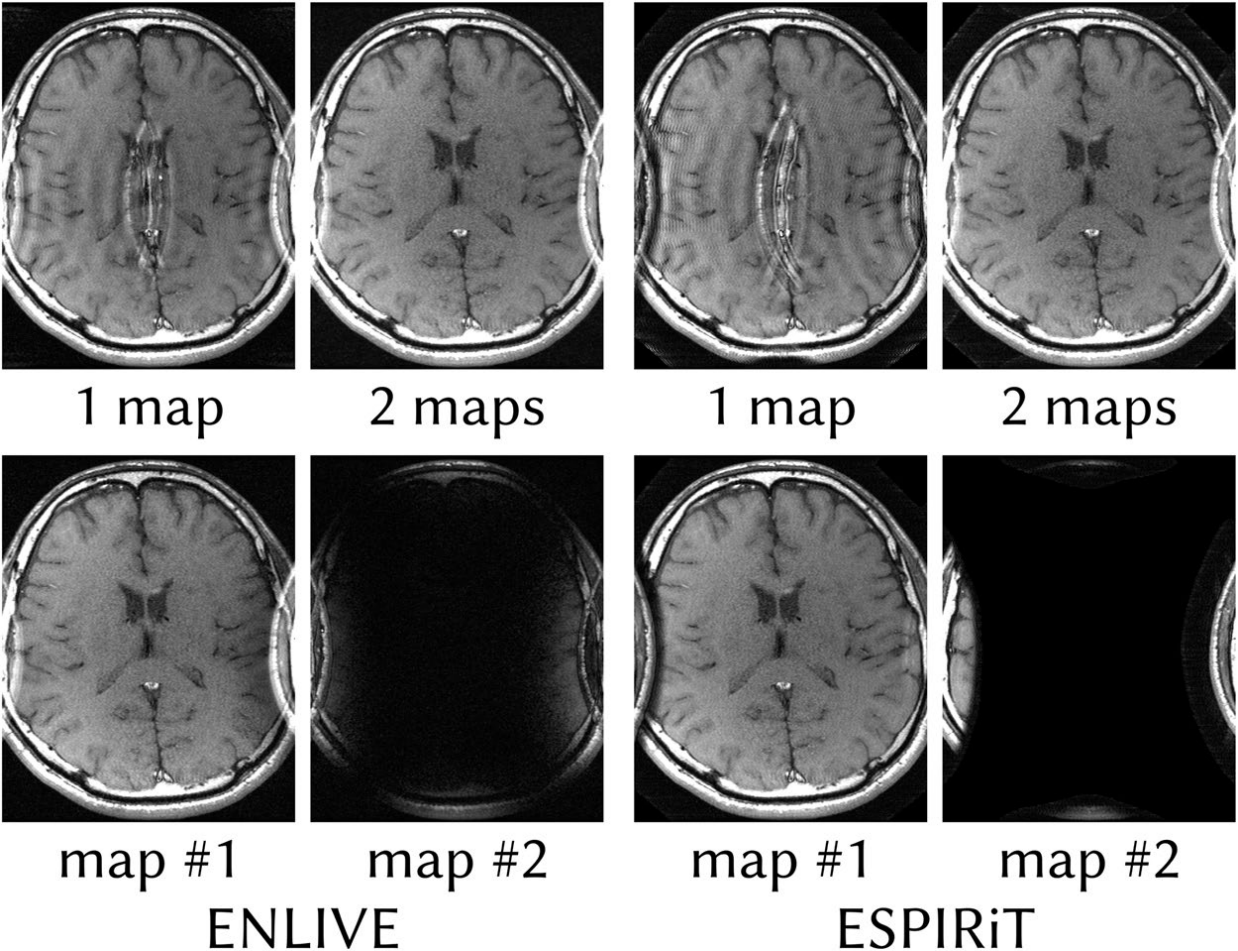
Comparison of ENLIVE and ESPIRiT reconstructions allowing both one and two sets of maps (top row) together with individual map images (bottom row) for the reconstructions using two maps. While the reconstructions using a single set of maps exhibit strong aliasing artifacts, the reconstructions allowing two sets of maps are artifact-free. The reason can be seen in the individual images: A single image with a single set of coil profiles cannot resolve the aliasing arising from the infolded sides. Using two sets of maps, the region causing infolding can be separated into the second image.

**Figure 2:**
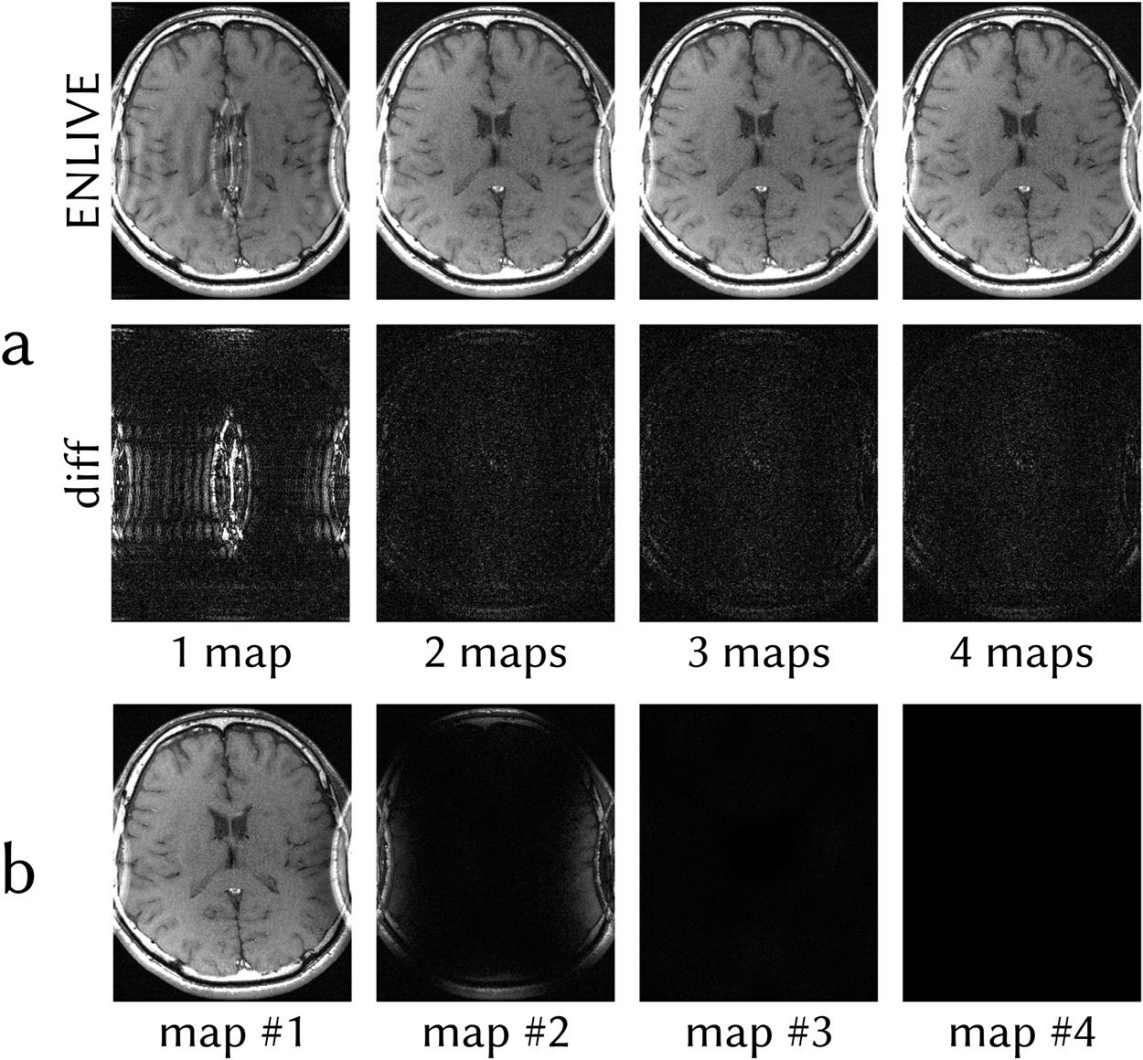
(**a**) ENLIVE reconstructions of the same data as in Fig. 1 using 1, 2, 3 and 4 sets of maps. Difference images to fully-sampled reference data are shown in the bottom row. Using a single map, the central artifact is clearly visible in the reconstruction as well as in the difference image. Using 2 and more maps, the artifact is resolved and the difference images show close to no variation. (**b**) Individual map images of the reconstruction using 4 maps. Since 2 sets of maps are sufficient to fully describe the data, the first two maps are similar to the maps depicted in Fig. 1 while maps 3 and 4 are close to zero. The corresponding coil profiles are depicted in Fig. 3.

**Figure 3:**
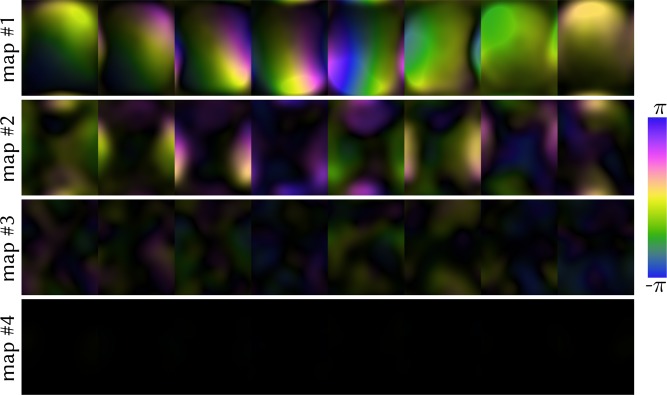
Calculated coil sensitivity profiles for the ENLIVE reconstruction using 4 sets of maps shown in Fig. 2. The second map is sensitive in the region which causes infolding in the single-map reconstruction, while the first map is smoothly sensitive over the entire FOV. The third and fourth map show very little sensitivity anywhere. Magnitude is encoded in brightness while phase in encoded in the color, according to the cyclic magenta-yellow-green-blue colormap described in^44^.

**Figure 4:**
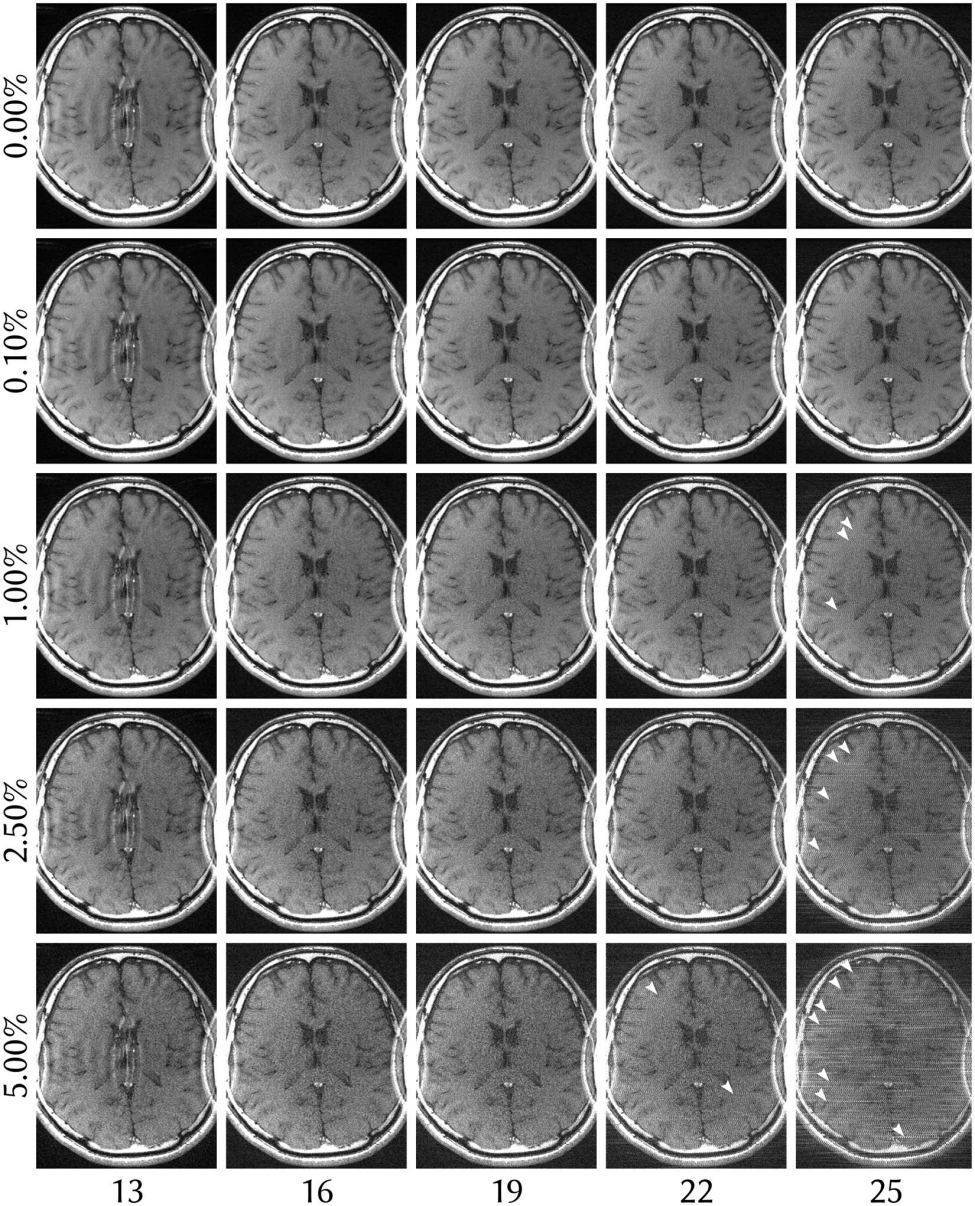
ENLIVE reconstruction with 2 maps with differing number of Newton steps (left to right) and different levels of added noise (top to bottom) of the same dataset as in Fig. 1. Gaussian white noise was added to the k-space before reconstruction. The standard deviation of the added noise was varied between 0 and 5% of the absolute value of the DC component. Using too few Newton steps leads to residual infolding artifacts, while too many Newton steps cause high-frequency artifacts to appear (some of which are indicated by arrows). Since the number of Newton steps controls the regularization in the IRGNM, we can understand these two effects as too much and too little regularization. In all cases, the added noise has no impact on the infolding artifact.

**Figure 5:**
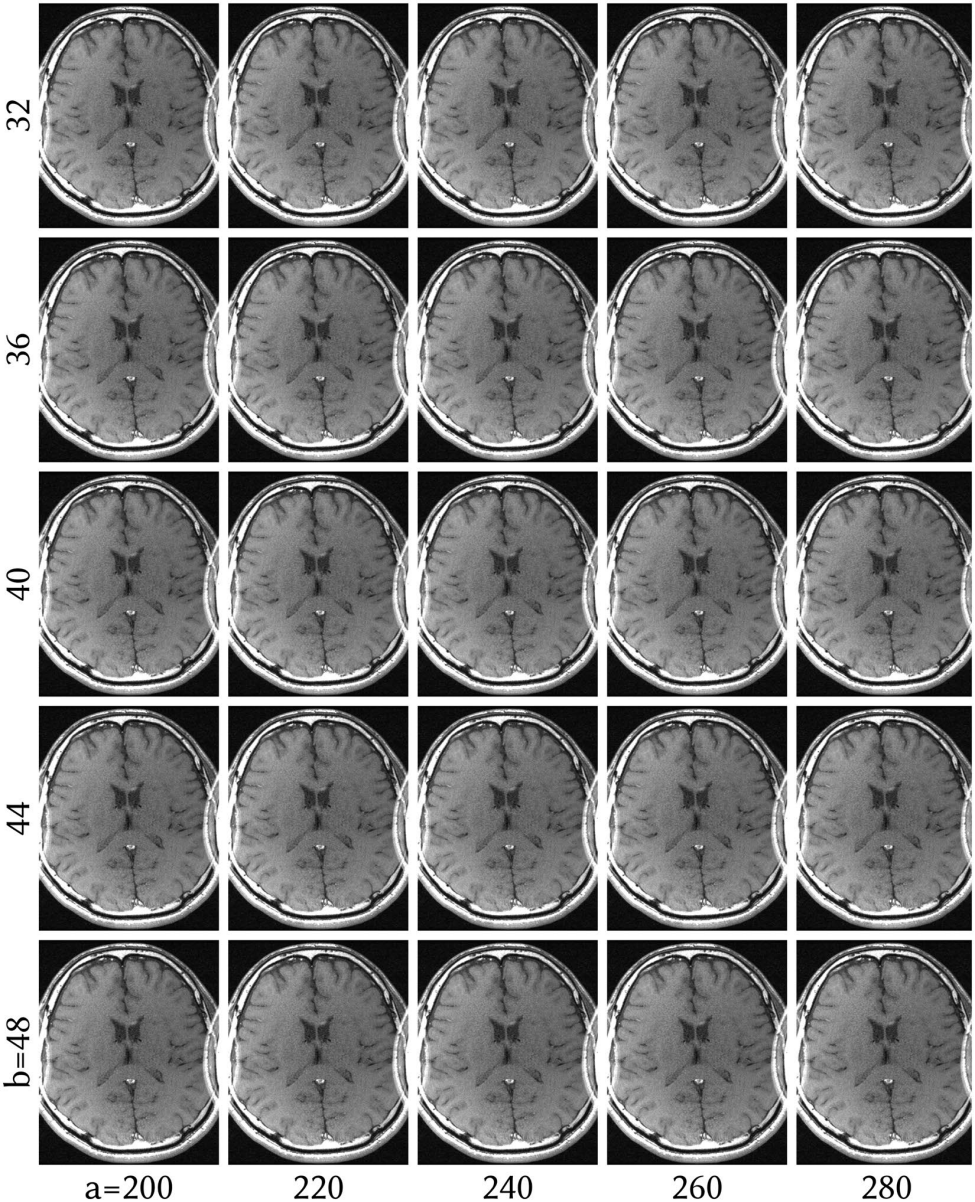
ENLIVE reconstruction with 2 maps of the same dataset as in Fig. 1 with different parameters for the coil weighting matrix ***W***. ***W*** applies a penalty in k-space according to ${(1+a{\Vert {\boldsymbol{k}}\Vert }^{2})}^{b/2}$. *a* varies from left to right while *b* varies from top to bottom. For all other reconstructions, *a* = 240 and *b* = 40 (center image) were used. The infolding artifact does not appear for any parameter pair, indicating that the reconstruction is not sensitive to specific values of *a* or *b*.

In the examples with a restricted FOV, both ENLIVE with a single set of maps, i.e. NLINV, and ESPIRiT reconstructions show a similar central artifact (Fig. 1). This artifact can be readily explained as a consequence of the undersampling pattern and the signal model violation at the edges of the image: Without a parallel imaging reconstruction, we expect aliasing artifacts from all pixels in the FOV. The parallel imaging reconstruction using a single set of maps can resolve this aliasing only for pixels outside of the regions of model violation. Since these edge regions alias to the image center, the artifact appears there. Both ENLIVE and ESPIRiT reconstructions allowing multiple sets of maps (Figs 1 and 2a) can resolve the aliasing everywhere. For ENLIVE, the coil profiles (Fig. 3) of the second map are sensitive in these regions. For ENLIVE using more than 2 sets of maps, the third and fourth map are close to zero (Fig. 2b). Since no thresholding is used, they cannot be exaclty zero. As is common in parallel imaging, tuning of the regularization is necessary for successful reconstruction: Fig. 4 shows that using too high regularization (too few Newton steps) does not eliminate the central infolding artifact, while too low regularization (too many Newton steps) leads to high-frequency artifacts. Added noise degrades image quality, especially in the case of too low regularization, but does not change the appearance of the infolding artifact. Additionally, Fig. 5 shows that the reconstruction is not sensitive to specific choices for the parameters *a* and *b* of the coil weighting matrix ***W***.

### Phase-constrained Imaging

**Figure 6:**
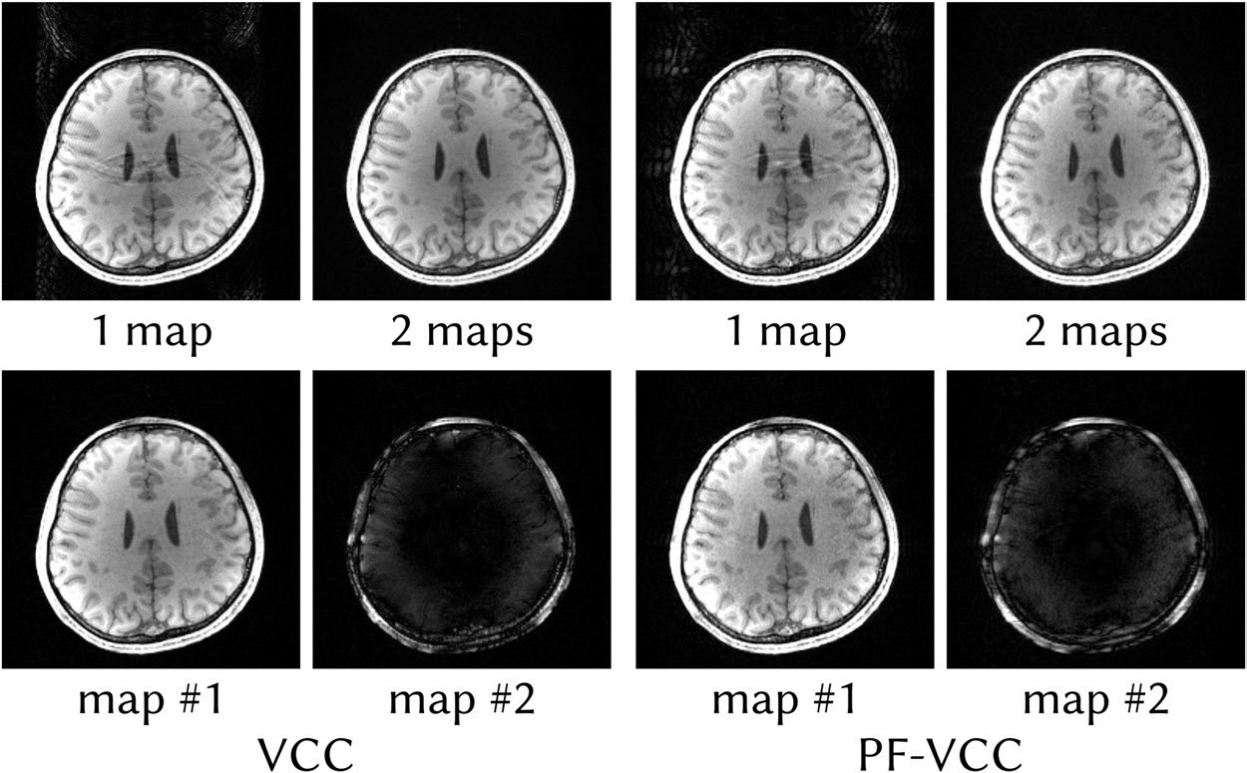
ENLIVE reconstructions allowing one and two sets of maps of data extended with virtual conjugate coils (VCC) and such data with a partial k-space (PF-VCC). The virtual-conjugate coils impose a real-value constraint onto the data. High-frequency phase close to the skull violates this constraint, leading to artifacts in reconstructions using a single set of maps. By allowing two sets of maps, these regions with high-frequency phase variation are separated into the second image, allowing almost artifact-free reconstruction.

Next, reconstructions for phase-constrained imaging using virtual-conjugate coils with and without an additional partial-Fourier factor are shown in Fig. 6. In both cases, reconstruction using only a single set of maps exhibit aliasing artifacts. These are a consequence of the real-value constraint imposed by using virtual-conjugate coils together with high-frequency phase variations caused by off-resonance from fat: A single real-valued image cannot account for this high-frequency phase, therefore the aliasing cannot be resolved. Relaxing the reconstruction by allowing multiple sets of maps resolves this problem, since the second set of maps can now account for this high-frequency phase variation.

### Phase Singularities

**Figure 7:**
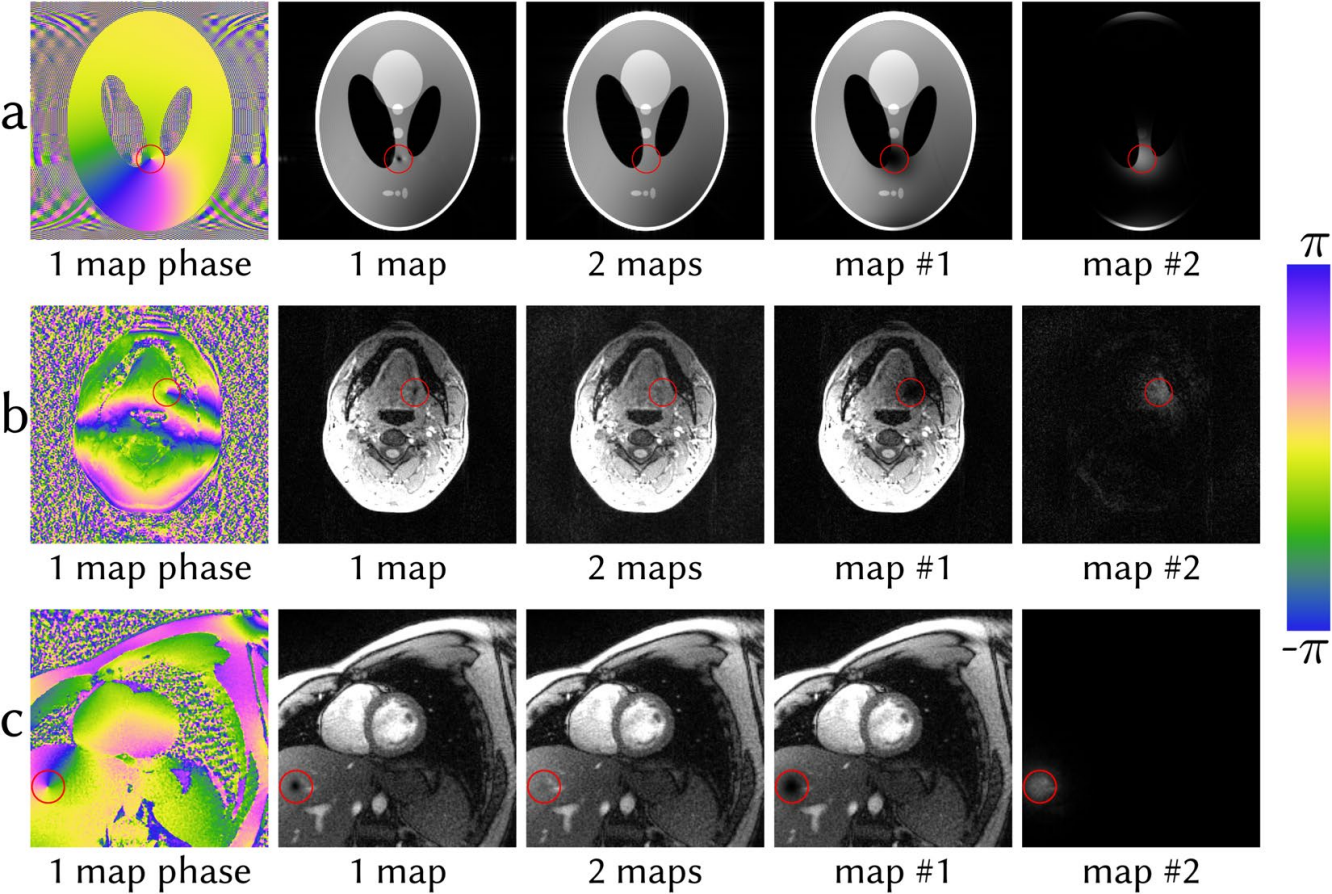
Phase singularities in (**a**) a numerical phantom, (**b**) a transversal slice through the lower jaw and (**c**) a non-Cartesian short axis-view of the human heart. Each dataset has been reconstructed with ENLIVE allowing one and two sets of maps. The phase singularity in (**a**) was produced by providing an initial guess containing a singularity. This singularity, clearly visible in the phase image, leads to artifactual signal loss at the same position in the post-processed magnitude image. As in (**a**), the phase singularities in (**b**,**c**) lead to signal loss at the corresponding positions in the magnitude images. By allowing two sets of maps, ENLIVE can resolve this artifact by using the second set of sensitivities around the phase singularity, thereby providing an artifact-free combined image.

Figure 7a shows a phantom example where the initial guess has been intentionally chosen to induce a phase singularity in the reconstruction. The phase singularity leads to signal loss using a single set of maps. Using ENLIVE allowing multiple sets of maps, the affected region can be resolved in the second map. By combining the images, a single image without signal loss can be recovered. This situation can also occur in practice. Figure 7b shows a slice through the throat with large phase variations, while Fig. 7c shows a short-axis view of the human heart acquired with radial FLASH. Using ENLIVE allowing multiple sets of maps, it is possible to reconstruct artifact-free images.

### Low-rank Property

**Figure 8:**
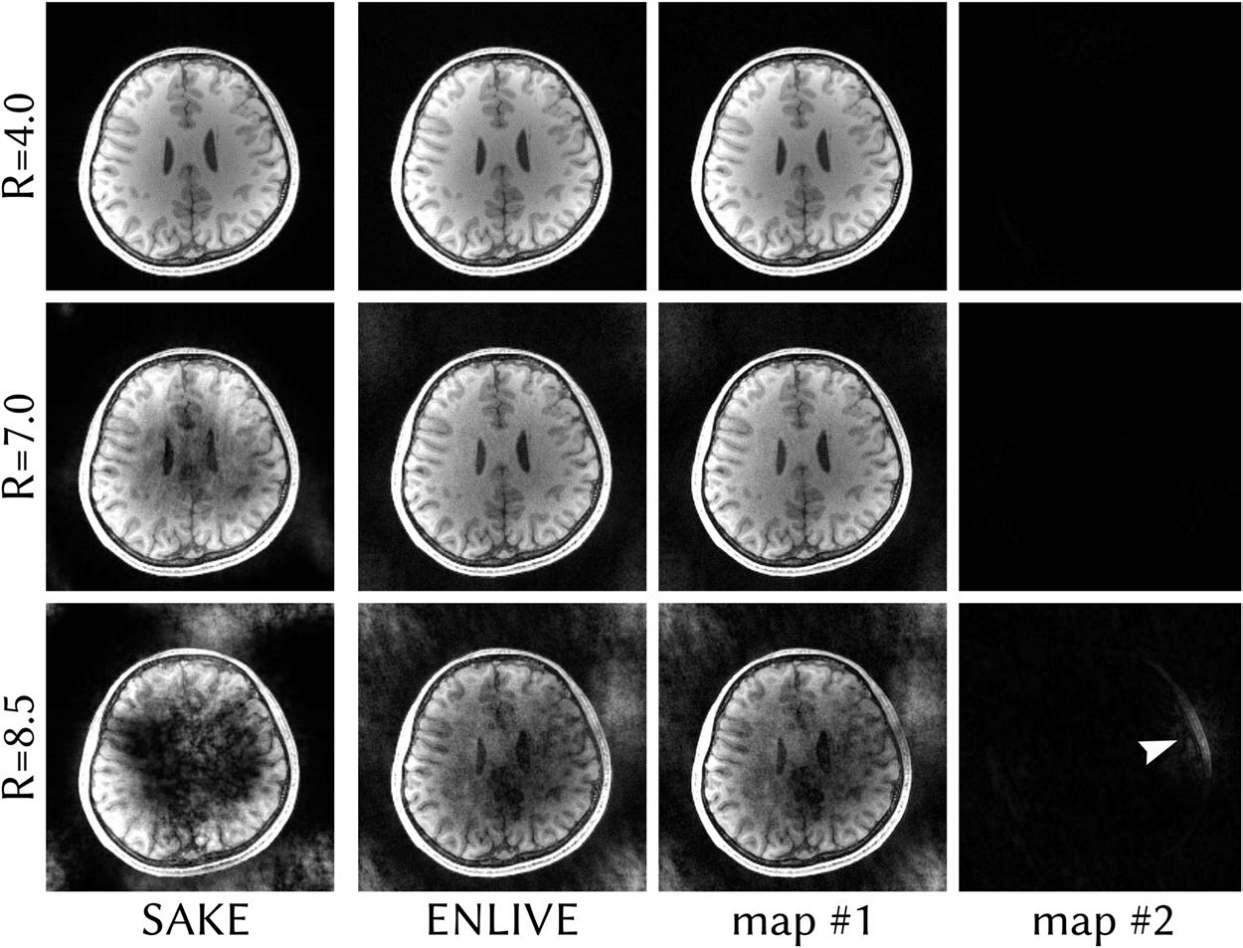
Variable-density Poisson-disc undersampled data with varying undersampling factors reconstructed with ENLIVE allowing two sets of maps and with SAKE. The same slice as in Fig. 6 is used. Since this is a calibrationless parallel imaging reconstruction without additional constraints and without model violations, a single set of maps is sufficient. For undersampling factors up to R = 7.0, ENLIVE therefore leaves the second allowed set empty, which causes the combined image to be essentially identical to the first set image. For an undersampling factor of R = 8.5, the ENLIVE reconstruction becomes very noisy and some image features start appearing the second map (indicated by an arrow). For R = 4.0, SAKE, too, provides artifact-free reconstruction. With higher undersampling factors artifacts appear in the images.

**Figure 9:**
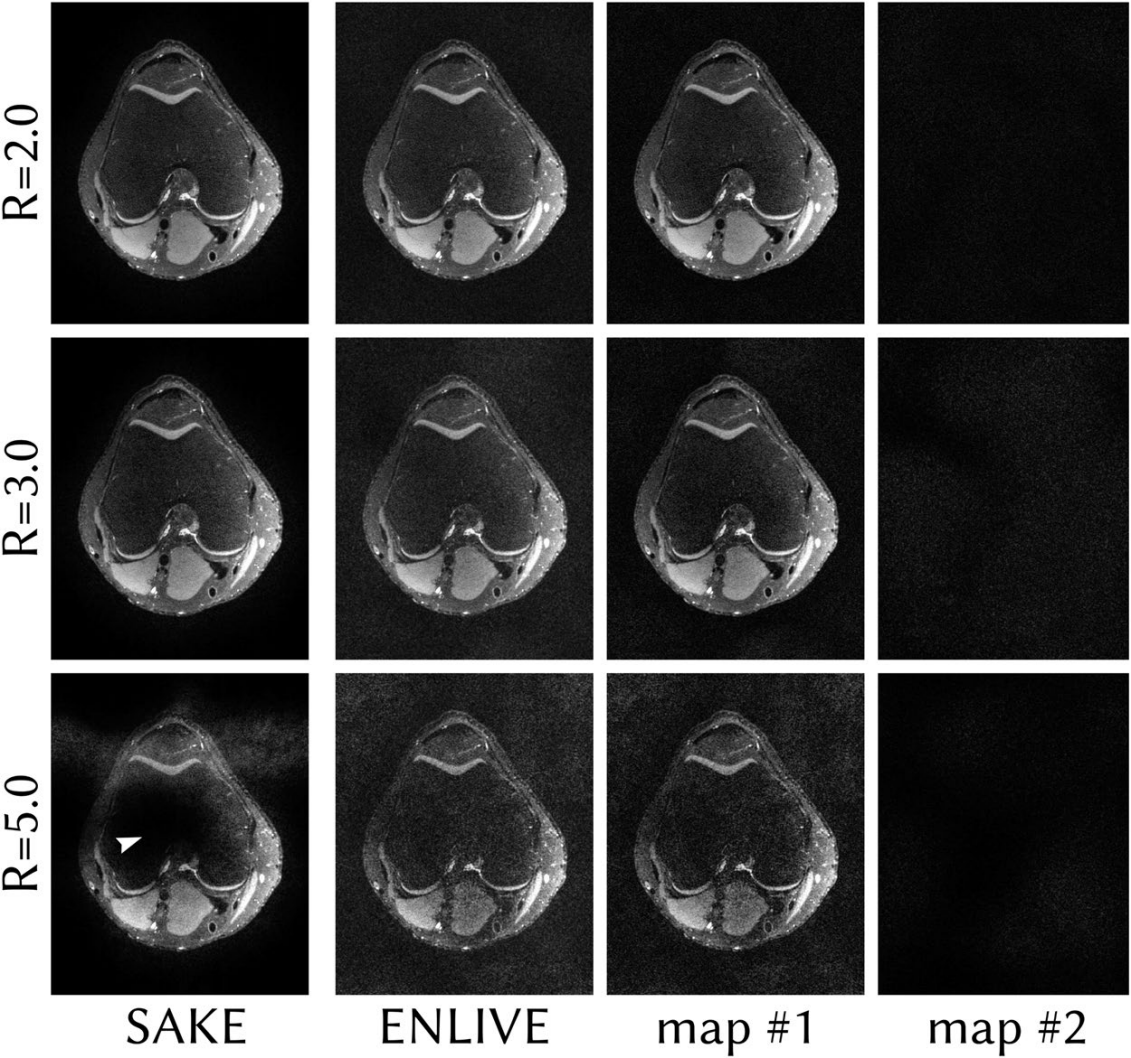
Variable-density Poisson-disc undersampled data of a human knee with varying undersampling factors reconstructed with ENLIVE allowing two sets of maps and with SAKE. This, too, is a dataset without model violations. The second ENLIVE is therefore close to zero. Up to R = 3.0, both SAKE and ENLIVE provide artifact free reconstruction. For R = 5.0, ENLIVE provides a reconstruction with high noise. SAKE, however, produces a large signal void in the image center (indicated by an arrow).

Figures 8 and 9 show calibrationless variable-density Poisson-disc undersampled reconstructions with differing undersampling factors comparing ENLIVE to SAKE. In Fig. 8, both ENLIVE and SAKE provide artifact-free reconstruction for moderate undersampling up to *R* = 4.0. At *R* = 7.0, SAKE shows artifacts while ENLIVE is artifact free. For these undersampling factors, the second ENLIVE set image is close to zero, while the first set contains the image. For *R* = 8.5, both ENLIVE and SAKE show strong artifacts. Additionally, the second ENLIVE map shows some image features. Reconstruction time for *R* = 4.0 for this dataset using a single core of an Intel Core i5-4590 CPU was 22 s using ENLIVE and 6.3 h using SAKE. In Fig. 9, ENLIVE and SAKE provide artifact-free reconstruction up to *R* = 3.0. At *R* = 5.0, ENLIVE reconstruction is noisy while SAKE shows a large signal void. Reconstruction time for *R* = 2.0 for this dataset using a single core was 18.6 s using ENLIVE and 41.5 min using SAKE.

**Figure 10:**
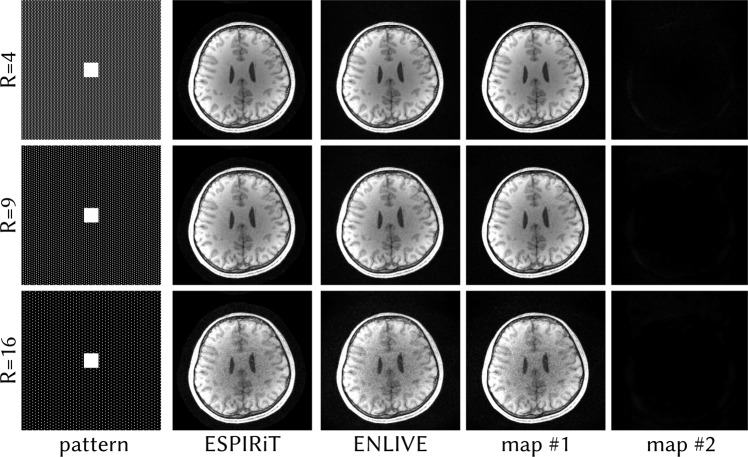
Comparison of ENLIVE reconstruction using 1 and 2 maps and ESPIRiT reconstruction using 2 maps of the same dataset as in Fig. 6 undersampled with Cartesian CAIPIRINHA patterns with differing undersampling factors. Using two maps, ENLIVE and ESPRiT reconstructions show comparable quality. Even though noise is increasing with higher undersampling, the second map remains close to zero. We conjecture that the adequate calibration region inhibits undersampling artifacts and ensures that no signal appears in the second map, in contrast to Fig. 8.

Figure 10 shows Cartesian ENLIVE reconstructions of data undersampled using CAIPIRINHA patterns with different undersampling factors. As a reference, the corresponding patterns are shown in the first column. For all undersampling factors, the second map image is close to zero wile the first map contains the entire image. With increasing undersampling, high noise starts to appear in the first map and the combned image. Still, no undersampling artifacts appear even at *R* = 16. Furthermore, even at this high undersampling, no image features appear in the second map, in contrast to the result in Fig. 8. We conjecture that the adequate calibration region in this datasets prevents that artifact.

## Discussion

This work introduces ENLIVE, a nonlinear reconstruction method for parallel imaging using a relaxed forward model. Using the IRGNM, ENLIVE simultaneously estimates multiple sets of images and coil sensitivity profiles, extending NLINV by ESPIRiT’s approach of using multiple sets of maps. The resulting bi-linear problem with ${\ell }_{2}$-regularization can be related to a lifted linear formulation using nuclear norm regularization, which promotes low-rank solutions. From this, it becomes apparent that the method, while employing a different parametrization, is similar to SAKE and P-LORAKS^6,7^, which are based on structured low-rank matrix completion in k-space, and to CLEAR^8^, which locally promotes low-rankness in the image domain. Although the low-rankness of the matrix considered in the k-space methods is also caused by the fact that the signal lives in a sub-space spanned by the coil sensitivities^5,9^, it is constructed from many shifted copies of the signal in k-space. This leads to a huge linear reconstruction problem with a rank constraint. In contrast, CLEAR uses block-wise reconstruction in the image domain, which is more similar to ENLIVE, but still requires a large number of small SVDs. A similar concept has been used to implement other low-rank methods. For example, building on top of the work on object modeling introduced in^17^, several approaches using annihilating filters have recently been proposed for combining parallel imaging with compressed sensing^18–21^. The existence of annihilating filters implies in turn the existence of a weighted low-rank Hankel matrix which can be constructed from the k-space samples. These methods then recover missing samples by structured low-rank matrix completion. In ENLIVE, the convex matrix completion problem has been replaced by a much smaller bi-linear problem with simple quadratic penalties^15,16^. In some sense, this is similar to the idea of transforming linear problems with ${\ell }_{1}$-regularization into quadratic problems with ${\ell }_{2}$-regularization^22^.

Low-rank approaches have also been proposed for dynamic imaging. One method for blind compressed sensing^23^ estimates both the time series of images as well as a dictionary which sparsifies that series. Haldar and Liang^24^ introduce a method which uses partial separability of the signal into functions describing its k-space and its time dependence. Both of these approaches exploit the low rank of the time-dependent signal. While structurally similar, Haldar and Liang^24^ use an explicitly rank-constraint formulation while Lingala *et al*.^23^ use an ${\ell }_{1}$-norm to induce sparsity. In contrast, ENLIVE’s ${\ell }_{2}$-regularization achieves low-rankness even below its constraint on the maximum rank through the equivalence to a formulation with regularization of the nuclear norm outlined in the Theory, which forms the core of the proposed method.

ENLIVE can also be related to a previous extension of NLINV proposed for separation of chemical species^25,26^. This method is based on the idea that the signal is a superposition of different images shifted in the spatial domain according to the chemical shift. As also shown for ESPIRiT, the sensitivities for the shifted signals from different species also appear to be shifted. They therefore violate the simple SENSE model with a single set of of maps and, consequently, cause the appearance of a second set of maps. The previously proposed extension to NLINV can be understood as a version of ENLIVE with the additional constraint that different sets of sensitivities are shifted versions of each other.

As shown in this work, small FOV and phase-constrained reconstructions using a single set of maps show artifacts whenever there are inconsistencies which cannot be explained using the simple model, while ENLIVE allowing two sets of maps enables artifact-free reconstruction in all evaluated cases. When using correct regularization, added noise does not impede artifact removal either. In cases where reconstruction with a single set of maps is already free from artifacts, ENLIVE automatically only uses a single set. In general, though, the maximum number of ENLIVE maps must be specified manually. This is similar to ESPIRiT where, while theoretically the correct number of maps can automatically be estimated as the multiplicity of the eigenvalue 1, in practice a maximum number of maps is set in advance to enable efficient computation of the eigenvector maps by power iteration. However, an extension to ENLIVE to automatically adapt the number of maps during the iteration is also conceivable.

As the distribution of the phase between image and coil sensitivities cannot be determined from the data alone without additional prior knowledge, choosing a good phase is a common problem when calibrating sensitivities^27,28^. This fundamental problem affects different algorithms in different ways. In Walsh’s method^29^ or ESPIRiT a pixel-wise phase across channels simply remains undefined and has to be aligned to a reference. If the reference is not ideal, phase singularities may occur. Phase singularities imply a non-smooth phase which then reduces sparsity in compressed sensing, preventing an efficient and compact representation of the sensitivities in the Fourier domain^30^, or causing problems in post-processing. For example, as Li *et al*.^31^ have shown, phase singularities can appear as artifactual microhemorrhage in susceptibility weighted imaging. NLINV and ENLIVE guarantee smooth sensitivities, but this then traps the algorithm in a local minimum and creates a hole instead^32^. For ENLIVE, the use of a second set of maps may still avoid signal loss in the reconstruction.

Even though local minima are a general concern with nonlinear methods, in our experience, the only practically relevant examples are the phase singularities. There, although the ENLIVE reconstruction is not optimal, use of a second map may mitigate the resulting artifact.

Compared to ESPIRiT, ENLIVE is more flexible since it has fewer prerequisites for its use, e.g. no calibration region is necessary. However, in the case of an undersampled Cartesian acquisition with calibration region, ESPIRiT is still to be preferred in most cases because of its speed. Only when faced with a very large number of channels might ESPIRiT lead to longer reconstruction times due to the unfavorable scaling of its SVD with the number of channels.

In summary, ENLIVE combines different advantages of NLINV, ESPIRiT, and SAKE. As NLINV and SAKE, it utilizes all available data, can be directly applied to non-Cartesian data, and does not require a calibration region. As ESPIRiT and SAKE, it is not limited to the SENSE model but automatically adapts to certain inconsistencies in the data. As ESPIRiT and NLINV, it is computationally efficient and makes use of an explicit image-domain representation during reconstruction which facilitates the use of advanced regularization terms.

## Conclusion

In this work we propose ENLIVE, a nonlinear method for parallel imaging which seeks to combine the robustness of ESPIRiT with the flexibility of NLINV. ENLIVE can be related to a lifted formulation of blind multi-channel deconvolution with nuclear norm regularization, which show that it belongs to the class of calibrationless parallel imaging methods based on structured low-rank matrix completion. In imaging settings involving limited FOV, phase constraints, and phase singularities, it has been shown to provide artifact-free reconstruction with quality comparable to state-of-the-art methods.

## Methods

The proposed method was implemented in the Berkeley Advanced Reconstruction Toolbox (BART)^33^ and all other reconstructions were performed using BART as well. Process-level parallelization was achieved using GNU parallel^34^. To facilitate the reproducibility of our research, data and source code used to generate the results of this paper can be downloaded from https://github.com/mrirecon/enlive.

To test its robustness in case of inconsistencies, ENLIVE was applied in several different experimental settings: We selected examples for imaging with an FOV smaller than the extent of the object, phase-constrained imaging, and phase singularities. In all cases, reconstructions using ENLIVE were performed using one, i.e. NLINV, or two sets of maps with initial regularization set to *α*_0_ = 1. If not stated otherwise, 11 Newton steps and *q* = 1/2 were used for the IRGNM. These parameters, as well as the parameters for the other methods, were chosen according to best visual appearance.

All volunteer imaging for this study was performed with their prior informed written consent, in accordance with the relevant guidelines and regulations, and with the approval of the ethics committee of the University Medical Center Göttingen.

In an example without inconsistencies we tested whether ENLIVE produces results with only one set of maps. Additional examples show ENLIVE’s performance under high undersampling and in non-Cartesian imaging.

### Limited FOV

We applied ENLIVE to the same dataset used in^9^. This is a retrospectively 2-fold undersampled 2D spin-echo dataset (TR/TE = 550/14 ms, FA = 90°, BW = 19 kHz, matrix size: 320 × 168, slice thickness: 3 mm, 24 × 24 calibration region) with an FOV of 200 × 150 mm^2^, acquired at 1.5 T using an 8-channel head coil. The dataset was zero-padded in k-space to produce square image space pixels. This FOV is smaller than the head of the subject in the lateral direction which leads to artifacts in a traditional SENSE reconstruction. These data were reconstructed with ENLIVE using one or two sets of maps and compared to ESPIRiT using one or two sets of maps. To investigate the effect of additional sets of maps, the data were additionally reconstructed using 1, 2, 3, and 4 sets of maps. For ENLIVE, *q* = 2/3 was used. To investigate the sensitivity to noise and to regularization, an additional reconstruction using 13, 16, 19, 22 and 25 Newton steps and added Gaussian white noise with noise levels of 0%; 0.1%; 1%; 2.5%; 5% was performed. The noise level here is the standard deviation of the added noise as percent of the magnitude of the DC component. From this, 19 Newton steps was determined as the optimum and used for reconstruction. For ESPIRiT a kernel size of 6 × 6 and a threshold of 0.001 was used.

### Phase-constrained Imaging

Phase-constrained parallel imaging^35^ with virtual conjugate coils^36^ is equivalent to an explicit phase constraint in SENSE, but more robust in GRAPPA and ESPIRiT due to their ability to adapt to inconsistencies^11,37^. To assess ENLIVE’s performance in phase-constrained imaging settings with virtual conjugate coils, we applied it to the same dataset used in^11^. This is a single slice in readout direction of a retrospectively 3-fold undersampled 3D FLASH dataset (TR/TE = 11/4.9 ms) acquired at 3 T using a 32-channel head coil. 24 × 24 auto-calibration lines were used. Additionally, a partial Fourier factor of 5/8 was applied to the data and evaluated separately.

### Phase Singularities

Similar to other algorithms^27,28,32^ phase singularities can appear in coil sensitivity profiles with ENLIVE. As ENLIVE enforces smooth coil sensitivity profiles, this leads to an artifactual hole in the sensitivities around the singularity. To demonstrate this effect, we synthetically constructed an example using BART to generate 6-channel k-space data (matrix size: 256 × 256) of the numerical Shepp-Logan phantom. To get ENLIVE trapped in a local minimum with a phase singularity, we provided an initial guess already containing a phase singularity. In regions with rapid phase variation, such phase singularities can also appear in ENLIVE reconstructions of *in-vivo* data. A transversal slice through the throat containing such a phase singularity was selected from the same dataset used for phase-constrained imaging.

To further show that ENLIVE can be applied directly to non-Cartesian data, we reconstructed selected data containing a phase singularity from a real-time FLASH^38^ acquisition using a 30 channel thorax coil of a short-axis view through the heart of a volunteer with no known illnesses (TR/TE = 2.22/1.32 ms, FA = 10°, matrix size: 160 × 160, FOV = 256 × 256 mm^2^, slice thickness: 6 mm, field strength: 3.0T). Five consecutive frames during diastole, comprising 65 radial spokes, were selected, corrected for gradient delays^39^, regridded to a 1.5 times finer grid and subsequently reconstructed with ENLIVE using 1 and 2 maps. For this dataset, *q* = 2/3 and 17 iterations of the IRGNM were used.

### Low-rank Property

In order to show that ENLIVE automatically uses only the required number of sets of maps, we retrospectively undersampled the same 3D dataset used for phase-constrained imaging using variable-density Poisson-disc sampling^40^ with undersampling factors of *R* = 4.0, 7.0, 8.5 and without a calibration region, and then extracted the same slice in readout direction. As a comparison, these data were also reconstructed using SAKE with 50 iterations and a relative size of the signal subspace of 0.05.

Additionally, we applied SAKE and ENLIVE to a 3D fast spin-echo acquisition^41^ of a human knee (TR/TE = 1550/25 ms, FA = 90°, echo train length: 40, matrix size: 320 × 256, FOV = 160 × 153.6 mm^2^, field strength: 3.0T) from mridata.org^42^. This dataset was also undersampled using variable-density Poisson-disc sampling with undersampling factors of *R* = 2, 3, 5 and a single slice in readout direction was extracted. These data were then reconstructed using ENLIVE with 1 and 2 maps and with SAKE with 50 iterations and a relative size of the signal subspace of 0.125.

To evaluate ENLIVE in settings with high acceleration factors, we undersampled the 3D dataset used for phase-constrained imaging using Cartesian CAIPIRINHA^43^ patterns with undersampling factors of *R* = 4, 9, 16 with a 24 × 24 calibration region. These data were then reconstructed with ENLIVE using 2 maps with *q* = 1/3 and 8 iterations of the IRGNM.

## Supplementary information

Appendix to: ”ENLIVE: An Efficient Nonlinear Method for Calibrationless and Robust Parallel Imaging”
