# Supplementary material for: ENLIVE: An Efficient Nonlinear Method for Calibrationless and Robust Parallel Imaging
Source: Sci Rep. 2019 Feb 28;9:3034. doi: 10.1038/s41598-019-39888-7 (PMC6395635; doi:10.1038/s41598-019-39888-7)
Supplement: Supplementary file 1 — Appendix to: ”ENLIVE: An Efficient Nonlinear Method for Calibrationless and Robust Parallel Imaging” [file 41598_2019_39888_MOESM1_ESM.pdf]

Appendix to: ”ENLIVE: An Efficient Nonlinear Method for Calibrationless and Robust Parallel Imaging”

H. Christian M. Holme[1,2,*], Sebastian Rosenzweig[1,2], Frank Ong[3], Robin N. Wilke[1,2], Michael Lustig[3], and Martin Uecker[1,2]

[1]Institute for Diagnostic and Interventional Radiology, University Medical Center Göttingen, Göttingen, Germany
[2]German Centre for Cardiovascular Research (DZHK), Partner site Göttingen, Göttingen, Germany
[3]Departement of Electrical Engineering and Computer Sciences, University of California, Berkeley
[*]christian.holme@med.uni-goettingen.de

Appendix

Operator $\mathcal{A}$

Here, we show the layout of $\boldsymbol{u}$, $\boldsymbol{v}$, and $\boldsymbol{u}\boldsymbol{v}^T$ as well as the action of $\mathcal{A}$ using an image of size $N_I := n_x \cdot n_y \cdot n_z$ and N_C coils. Then, the vector $\boldsymbol{u} \equiv \boldsymbol{m} \in \mathcal{C}^{N_I}$ is defined as

$$\boldsymbol{u}^T = \begin{pmatrix} m_1 & \dots & m_{N_I} \end{pmatrix} \tag{8}$$

and the vector $\boldsymbol{v} \in \mathcal{C}^{N_C \cdot N_I}$ of stacked, weighted coil sensitivity profiles $\hat{\boldsymbol{c}}_j$ as

$$\boldsymbol{v}^T = \begin{pmatrix} \hat{c}_{1,1} & \dots & \hat{c}_{N_I,1} & \hat{c}_{2,2} & \dots & \hat{c}_{N_I,N_C} \end{pmatrix} \tag{9}$$

where $\hat{c}_{ij}$ is the ith pixel of the jth weighted coil profile. Therefore, $\boldsymbol{u}\boldsymbol{v}^T \in \mathcal{C}^{N_I \times N_C \cdot N_I}$ is

$$\boldsymbol{u}\boldsymbol{v}^T = \begin{pmatrix} m_1\hat{c}_{1,1} & m_1\hat{c}_{2,1} & \dots & m_1\hat{c}_{N_I,1} & m_1\hat{c}_{1,2} & m_1\hat{c}_{2,2} & \dots & m_1\hat{c}_{N_I,N_C} \\ m_2\hat{c}_{1,1} & m_2\hat{c}_{2,1} & \dots & m_2\hat{c}_{N_I,1} & m_2\hat{c}_{1,2} & m_2\hat{c}_{2,2} & \dots & m_2\hat{c}_{N_I,N_C} \\ \vdots & \vdots & \ddots & \vdots & \vdots & \vdots & & \vdots \\ m_{N_I}\hat{c}_{1,1} & m_{N_I}\hat{c}_{2,1} & \dots & m_{N_I}\hat{c}_{N_I,1} & m_{N_I}\hat{c}_{1,2} & m_{N_I}\hat{c}_{2,2} & \dots & m_{N_I}\hat{c}_{N_I,N_C} \end{pmatrix} \tag{10}$$

Applying the inverse of the weighting matrix $\boldsymbol{W}$ yields

$$\boldsymbol{u}\boldsymbol{v}^T\boldsymbol{W}^{-1} = \begin{pmatrix} \boldsymbol{m_1 c}_{1,1} & m_1 c_{2,1} & \dots & m_1 c_{N_I,1} & \boldsymbol{m_1 c}_{1,2} & m_1 c_{2,2} & \dots & m_1 c_{N_I,N_C} \\ m_2 c_{1,1} & \boldsymbol{m_2 c}_{2,1} & \dots & m_2 c_{N_I,1} & m_2 c_{1,2} & \boldsymbol{m_2 c}_{2,2} & \dots & m_2 c_{N_I,N_C} \\ \vdots & \vdots & \ddots & \vdots & \vdots & \vdots & & \vdots \\ m_{N_I} c_{1,1} & m_{N_I} c_{2,1} & \dots & \boldsymbol{m}_{N_I}\boldsymbol{c}_{N_I,1} & m_{N_I} c_{1,2} & m_{N_I} c_{2,2} & \dots & \boldsymbol{m}_{N_I}\boldsymbol{c}_{N_I,N_C} \end{pmatrix} \tag{11}$$

The diagonals containing products of image pixels with corresponding coil profile pixels are highlighted in bold. The action of the operator $\mathcal{A}$ is to select these highlighted entries of $\boldsymbol{u}\boldsymbol{v}^T\boldsymbol{W}^{-1}$, apply a two or three dimensional Fourier transform to each coil image and finally apply a mask $\mathcal{P}$ projecting onto the acquired pattern.

Equivalence of formulations

In the following we will show that the lifted rank-k problem in Eq. (4) corresponds to the ENLIVE formulation in Eq. (2). Using the linearity of the operators, the following holds:

$$\mathcal{A}\left\{\boldsymbol{U}\boldsymbol{V}^T\right\} = \mathcal{A}\left\{\sum_{i=1}^{k} \boldsymbol{u}_i\boldsymbol{v}_i^T\right\} \tag{12}$$

$$= \sum_{i=1}^{k} \mathcal{A}\left\{\boldsymbol{u}_i\boldsymbol{v}_i^T\right\} \tag{13}$$

$$= \sum_{i=1}^{k} \left(P\mathcal{F}\left\{\boldsymbol{c}_j^i \odot \boldsymbol{m}^i\right\}\right)_{j=1\dots N_c} \tag{14}$$

$$= \left(P\mathcal{F}\left\{\sum_{i=1}^{k} \boldsymbol{c}_j^i \odot \boldsymbol{m}^i\right\}\right)_{j=1\dots N_c} \tag{15}$$

Here, we make use of the definition of the operator $\mathcal{A}$:

$$\mathcal{A}\left\{\boldsymbol{u}_i\boldsymbol{v}_i^T\right\} := \left(P\mathcal{F}\left\{\boldsymbol{c}_j^i \odot \boldsymbol{m}^i\right\}\right)_{j=1\dots N_c} \tag{16}$$
